# Chronic hepatitis c genotype-4 infection: role of insulin resistance in hepatocellular carcinoma

**DOI:** 10.1186/1743-422X-8-496

**Published:** 2011-11-01

**Authors:** Amal A Mohamed, Samah A Loutfy, James D Craik, Abdel Gawad M Hashem, Ibrahem Siam

**Affiliations:** 1 Biochemistry Department, National Hepatology and Tropical Medicine Institute, Cairo 11796, Egypt; 2 Virology and Immunology Unit, Cancer Biology Department, National Cancer Institute, Cairo University, Cairo 11796, Egypt; 3 Biochemistry Department, Faculty of Medicine, Health Sciences Center, Kuwait University, Kuwait; 4 Microbiology Department, Faculty of Pharmacy, Cairo University, Cairo 11562, Egypt; 5 Internal Medicine Department, National Research Center, Cairo, Egypt

**Keywords:** hepatitis C, HCV-4, insulin resistance, fibrosis, hepatocellular carcinoma

## Abstract

**Background:**

Hepatitis C virus (HCV) is a major cause of chronic hepatitis and hepatocellular carcinoma (HCC) and different HCV genotypes show characteristic variations in their pathological properties. Insulin resistance (IR) occurs early in HCV infection and may synergize with viral hepatitis in HCC development. Egypt has the highest reported rates of HCV infection (predominantly genotype 4) in the world; this study investigated effects of HCV genotype-4 (HCV-4) on prevalence of insulin resistance in chronic hepatitis C (CHC) and HCC in Egyptian patients.

**Methods:**

Fifty CHC patients, 50 HCC patients and 20 normal subjects were studied. IR was estimated using HOMA-IR index and HCV-4 load determined using real-time polymerase chain reaction. Hepatitis B virus was excluded by enzyme-linked immunosorbent assay. Standard laboratory and histopathological investigations were undertaken to characterize liver function and for grading and staging of CHC; HCC staging was undertaken using intraoperative samples.

**Results:**

HCC patients showed higher IR frequency but without significant difference from CHC (52% vs 40%, p = 0.23). Multivariate logistic regression analysis showed HOMA-IR index and International Normalization Ratio independently associated with fibrosis in CHC; in HCC, HbA1c, cholesterol and bilirubin were independently associated with fibrosis. Fasting insulin and cholesterol levels were independently associated with obesity in both CHC and HCC groups. Moderate and high viral load was associated with high HOMA-IR in CHC and HCC (p < 0.001).

**Conclusions:**

IR is induced by HCV-4 irrespective of severity of liver disease. IR starts early in infection and facilitates progression of hepatic fibrosis and HCC development.

## Introduction

Persistent Hepatitis C virus (HCV) infection is widespread; it affects millions of people worldwide and induces a range of chronic liver disease [1]. Chronic HCV infection causes progressive hepatic fibrosis and cirrhosis in up to 20% of patients and approximately 10%-20% of cirrhotic patients may go on to develop hepatocellular carcinoma (HCC) within five years [2]. HCC is the most frequent cause of death in patients infected with HCV, and epidemiological trends suggest that the mortality rate is rising [3]. Understanding the risk factors for HCC development in patients infected with HCV is thus of great importance for refinement of treatment strategies and healthcare delivery. HCV has a high mutation rate and six major genotypes have been characterized, each with a distinctive geographical distribution and pathological properties [1]. Egypt has the highest countrywide prevalence of HCV in the world; about 12 to 15% of the total population are infected [4], with HCV Genotype-4 (HCV-4) accounting for the overwhelming majority of HCV infections.

HCV has been identified as a cause of metabolic syndrome, a complex that includes dyslipidemia, diabetes and insulin resistance (IR). IR is a key feature of this syndrome and a variety of potential molecular pathways by which HCV may contribute to IR have been suggested [5]. Patients infected with HCV have significantly higher IR than healthy controls matched for age, sex and body mass index [6].

Recent studies have found that HCV-associated IR may cause; (i) hepatic steatosis, (ii) resistance to anti-viral treatment, (iii) hepatic fibrosis and esophageal varices, (iv) hepatocarcinogenesis and proliferation of HCC and (v) extrahepatic manifestations [7,8]. IR has emerged as a risk factor for a wide variety of cancers, including endometrial and breast (especially after menopause), colon, and rectal, esophageal, kidney, pancreatic, biliary, ovarian and cervical cancers [9]. In chronic HCV infection, IR can favor fibrosis progression directly and act indirectly by inducing steatosis in a genotype-dependent manner [10].

It has been reported that IR can increase the risk of developing HCC in patients with chronic HCV infection [11]. A multiplicity of viral and host factors may play a crucial role in facilitating the onset of IR in patients with chronic hepatitis C (CHC) that may ultimately end with HCC development [5]. Given high levels of endemic CHC infection in Egypt, and that IR is a potentially modifiable factor, a better understanding of correlations of IR with HCC among Egyptian patients infected with HCV-4 is urgently needed.

The focus of the study was to investigate: (i) the prevalence of IR among CHC and HCC patients and its possible role in HCC development in the context of high prevalence of HCV-4 infection and (ii) impact of other factors such as host characteristics (age, gender, etc.), viral parameters (viral load, viral genotype), and other variables including obesity and dyslipidemia on IR and hepatic carcinogenesis.

## Patients and Methods

### Patients

This prospective study was conducted with 120 participants divided into three groups. The first group comprised 50 patients with chronic hepatitis C genotype- 4 (CHC). The second group comprised 50 hepatocellular carcinoma (HCC) patients diagnosed and treated at Ain Shams University Specialized Hospital, Cairo, between February 2009 and February, 2010. A third group included 20 apparently healthy participants who had donated blood at the National Cancer Institute, Cairo University.

The Ethical Committee of Ain Shams University Specialized Hospital approved the study protocol, which was prepared in accordance with the ethical guidelines of the 1975 Declaration of Helsinki and later revisions. Written consent was obtained from all participants prior to enrollment in the study and all were mentally and physically capable of answering a questionnaire.

Inclusion criteria: adult patients of both sexes (20-70 years old), diagnosis within the previous 6 months, positive for HCV RNA in serum (by RT-PCR assay), with evidence of chronic hepatitis supported by liver biopsy. The control group was made from adults negative for HCV RNA. Patients were not receiving hepatitis treatment at the time of sampling.

The presence of HBV infection or co-infection was excluded by serum ELISA for anti-HBc and HBsAg. In addition to investigations needed to fulfill the selection criteria, all individuals included in this study were subjected to the following:

#### A. Medical History

Full history was taken with special reference to risk factors for liver diseases such as previous HCV exposure in surgical wards, blood transfusions, dental therapy, needlestick injury, history of HCV in the spouse and i.v. injection.

#### B. Physical Examination

Complete medical examination with particular focus upon the manifestations of hepatitis such as jaundice, hepatomegaly, and tenderness in the right hypochondrium. BMI was calculated as body weight in kilograms divided by the square of height in meters (kg/m^2^). Abdominal ultrasonography was performed for all patients.

#### C. Histopathological investigations

Liver biopsy specimens were formalin-fixed and paraffin embedded then sectioned and stained (hematoxylin and eosin) for routine histopathological examination. Grading and staging of chronic hepatitis was performed according to Modified Knodell's Score [12]. Liver tissues from HCC cases were collected intraoperatively. These samples were graded using World Health Organization (WHO) classification criteria [13] and staging was determined according to the American Joint Committee on Cancer [13].

#### D. Laboratory investigations

Venous blood samples were taken in the morning after 12-h overnight fast. Plasma glucose, HbA1c, serum alanine aminotransferase (ALT), aspartate aminotransferase (AST), gammaglutamyl transferase (GGT), albumin (Alb), total bilirubin levels (Bil), cholesterol (Chol), and triglycerides (TG) were measured by using Synchron CX4 clinical system (standard clinical laboratory methods) at the Clinical Laboratory Department, Ain Shams Specialized Hospital. Serum insulin levels and α-fetoprotein levels were estimated by serological techniques (Axyam System, Abbott Laboratories).

Platelet count (Plt) and Prothrombin Time (PT) measurements were performed for all patients; normal PT was 12 seconds (100% concentration and International Normalization Ratio (INR) of 1).

Insulin resistance (IR) was calculated on the basis of fasting levels of plasma glucose and serum insulin, according to the homeostasis model assessment (HOMA) method. Calculation for the HOMA model followed a standard protocol; insulin resistance (HOMA-IR) = fasting glucose (mg/dL) × fasting insulin (μIU/mL)/405 [14]. The HOMA-IR index has seen widespread use, with various cut-off values for insulin resistance. In many studies of Caucasian populations a cut-off value of 2.5 has been applied; other studies have used higher cut-off values, for example 3.0 [15]. Genetic variation with respect to different ethnic groups will influence choice of cut-off value (discussed by Esteghamati [16]); for this study we used a cut-off value of 4.0 [17].

#### E. Viral Markers

### ELISA assays

Sera of all patients and controls were tested for HBsAg, anti-HBc and anti-HCV antibodies by ELISA, using third generation kits (DiaSorin, Italy) according to the manufacturer's instructions.

### Polymerase chain reaction (PCR) for hepatitis C virus

RT-PCR was performed as previously described [18] and 10 μl samples of the amplicons were analyzed by electrophoresis (1.2% agarose gel, ethidium bromide staining).

### HCV genotyping

HCV genotype was determined using INNO-LiPAII and III versant Kit (Innogenetics, Ghent, Belgium) according to manufacturer's directions

### Quantitation of HCV-RNA in serum

HCV-RNA was quantitated in all patients' serum samples using Real Time PCR (RT-PCR) [15] (primers and RT-PCR reagents from Stratagene, Qiagen, USA). Low viremia was defined as viral load lower than 100 x10^3 ^IU/L, moderate viremia as viral load 100-1000 × 10^3 ^IU/L, and high viremia when viral load > 1000 x10^3 ^IU/L [19].

### Statistical analysis

Data was analyzed using SPSSwin statistical package version 15 (SPSS Inc., Chicago, IL). Chi-square test (Fisher's exact test) was used to examine relationships between qualitative variables. For quantitative data, comparison between two groups was undertaken using Mann-Whitney test and comparison between 3 groups with ANOVA test or Kruskal-Wallis test followed post-Hoc "Schefe test". Spearman-rho method was used to test correlation between numerical variables. Multivariate analysis was performed using multiple linear regression model using forward method for the significant factors affecting fibrosis on univariate analysis. Multivariate analysis (logistic regression) was performed to find the predictors for HCC development All tests are two-tailed; a p-value<0.05 was considered significant.

## Results

### Baseline characteristics

(Table 1): Ages of CHC patients (median 57 years; range 34-70 yrs) and HCC patients (median 60 years; range 35-76 yrs) were closely comparable, while control group participants were younger (median 35 years; range 19-57). Preponderance of males was observed with both CHC and HCC groups; 1:1.5 and 1:1.6 respectively. There was no significant difference between CHC and HCC patients regarding age or sex (p = 0.14 and 0.838, respectively). No significant difference was observed in BMI distribution between the study groups (p = 0.908).

**Table 1:** Demographic characteristics and laboratory parameters of the study groups; normal, chronic hepatitis C (CHC) and hepatocellular carcinoma (HCC)

Variables	Control N = 20	CHC N = 50	HCC N = 50	*P-value
**Age ***(Mean ± SD)*	34.1 ± 9.74	55.04 ± 9.58	58.8 ± 9.66	0.14
**Sex**				
Male: n = 69	8	30	31	
Female: n = 51	12	20	19	
M:F ratio	01:00.6	01:01.5	01:01.6	0.838
**Obesity N(%)**	8 (40%)	22 (44%)	20 (40%)	0.685
				
**LFT**				
***Median (Range)***				
ALT (IU/L)	29 (20-41)	60 (25-210)	64(34-103)	0.09
AST (IU/L)	31 (18-40)	60 (25-180)	120 (65-310)	**<0.001**
T. Bil (mg/dL)	0.8 (0.4-1)	1 (0.5-3.6)	2.2 (1.2-6)	**<0.001**
Albumin (g/dL)	3.9 (3.5-4.7)	3.6(1.9-4.2)	3 (1.6-3.4)	**<0.001**
INR	0.8 (0.7-0.9)	1.1(0.7-1.5)	1.2(1.1-1.5)	**<0.001**
GGT (IU/L)	29 (12-46)	56.5 (15-105)	190 (60-560)	**<0.001**
				
**Biochemical tests**				
***Median(range***),				
Fasting Glu (mg/dL)	99(80-142)	170(100-230)	188(76-890)	0.81
Fasting Ins (IU/mL)	3.6(2-5.3)	8(3.5-13.0)	9(3.6-15)	0.178
HbA1c (**% **)	4.2(2.7-6)	7(3.2-11.0)	8(4.2-13)	**0.053**
HOMA-IR	0.87(0.42-1.49)	3.42 (0.86-7.06)	4.18 (0.91-32.22)	0.643
Plt (x10^9^/L)	300(152-465)	150(102-300)	130(50-170)	**<0.001**
Cholesterol (mg/dL)	157(120-247)	190(160-270)	190(110-300)	0.954
TG (mg/dL)	153(125-254)	190(87-300)	190(120-300)	0.999
				
Serum AFP (ng/mL)	6 (2.9-10)	30 (5-125)	225 (150-1060)	**<0.001**
				
**HCV RNA**				
(viral load copies/IU/L) **N(%)**	no viremia			
Low** (n = 21)				
Moderate (n = 56)		19(38)	2(4)	**<0.001**
High (n = 23)		21(42)	35(70)	
				
**Stage of Fibrosis N(%)**		10(20)	13(26)	
I+II (n = 17)		16 (32)	1(2)	**<0.001**
III+IV+ V (n = 83)		34 (68)	49(98)	

Data are median (range), frequency (%); obesity defined as BMI>25; *P value: comparison between CHC and HCC patients. LFT, Liver function tests, ALT, alanine aminotransferase; AST, aspartate aminotransferase; INR, International normalization ratio (for blood clotting); GGT, gammaglutamyl transferase; Glu, glucose; Ins, insulin; HbA1c, glycosylated hemoglobin; HOMA-IR, Homeostasis Model for assessment of insulin resistance; Plt, platelet count; TG, triglycerides; AFP, alpha fetoprotein; **Viral load (copies/IU/L): Low (**<**100 × 10^3^), Moderate (100-1000 × 10^3^), High (>1000 × 10^3^)

### Biochemical, viral and pathological factors

HCC patients showed significantly higher median values of INR and HbA1c, than CHC patients (p < 0.0001, p = 0.05, respectively), but significantly lower median values for albumin and platelets (p < 0.001). HCC patients also showed significantly higher median values of AST, Bil, GGT and α-fetoprotein when compared to CHC patients (p < 0.001 for all). No significant differences were found between HCC and CHC patients regarding median values of ALT, glucose, fasting insulin, HOMA-IR index values, cholesterol and triglycerides (p = 0.09, = 0.81, = 0.178,= 0.643,= 0.954, = 0.999).

Frequency of high value for HOMA-IR index (>4) and presence of dyslipidemia were not significantly different between CHC and HCC patients (Table 2).

**Table 2:** Comparison of high risk variables between chronic hepatitis C (CHC) and hepatocellular carcinoma (HCC) patients

Variables	CHC N = 50 N(%)	HCC N = 50 N(%)	*Odds ratio*	P-value
Age				
>57years	20(40)	30(60)	0.44 (0.20-0.99)	0.04
Male gender	30(60)	31(62)	1.09(0.49-2.43)	0.84
Obese (BMI >25)	22(44)	20(40)	1.18(0.53-2.61)	0.68
**LFT **				
ALT (>37 IU/L)	46(92)	47(94)	0.73(0.16-3.46)	>0.99
AST (>40 IU/L)	37(74)	50(100)	1.35(1.15-1.59)	**<0.001**
T. Bil (> 1 mg/dL)	24(48)	50(100)	2.08(1.56-2.78)	**<0.001**
Albumin (>5.3 g/dL)	0	0		
INR (> 1)	32(64)	50(100)	1.56(1.27-1.92)	**<0.001**
GGT (>50 IU/L)	30(60)	50(100)	1.67 (1.33-2.09)	**<0.001**
				
**Biochemical tests**				
Fasting Glucose				
(>110 mg/dL)	48(96)	42(84)	4.57(0.92-22.73)	0.09
Fasting Insulin (>6.IU/mL)	37(74)	42(84)	0.54 (0.20-1.45)	0.22
HbA1c (>7%)	24(48)	35(70)	0.40(0.17-0.90)	**0.02**
HOMA-IR (>4)	20(40)	26 (52)	1.63 (0.74-3.59)	0.23
Plt (>400 × 10^9^/L)	0	0		-
Cholesterol (>200 mg/dL)	17(34)	20(40)	1.29 (0.57-2.92)	0.63
TG (> 150 mg/dL)	48(96)	47(94)	1.53(0.24-9.59)	>0.99
**Tumor marker**				
Serum AFP (>10 ng/mL)	30(12-57)	255(220-310)	26.00(6.68-101.20)	**<0.001**
				
**HCV RNA (viral load) N(%)**				
Moderate (100-1000 × 10^3^) n = 56	21(42)	35(70)	-	**<0.001**
Stage of Fibrosis				
III-V (n = 83)	34(68)	49(98)	23.06 (2.92-82.21)	**<0.001**

ALT, alanine aminotransferase; AST, aspartate aminotransferase; INR, International normalization ratio (for blood clotting); GGT, gammaglutamyl transferase; Glu, glucose; Ins, insulin; HbA1c, glycosylated hemoglobin; HOMA-IR, Homeostasis Model for assessment of insulin resistance; Plt, platelet count; TG, triglycerides; AFP, alpha fetoprotein;

^†^AFP is considered elevated if greater than 400 ug/mL according to international guidelines

HCC patients showed significantly higher frequency of presentation with moderate viral load than CHC patients (70% vs 42%, p < 0.001) and higher grades of fibrosis (IV and V: 45/50 (90%) for HCC vs 15/50 (30%), for the CHC group (p < 0.001).

The normal control group showed significant difference when compared to the HCC or CHC groups (p < 0.001) for all parameters studied, except BMI.

### Factors associated with development of fibrosis

**Figure 1:**
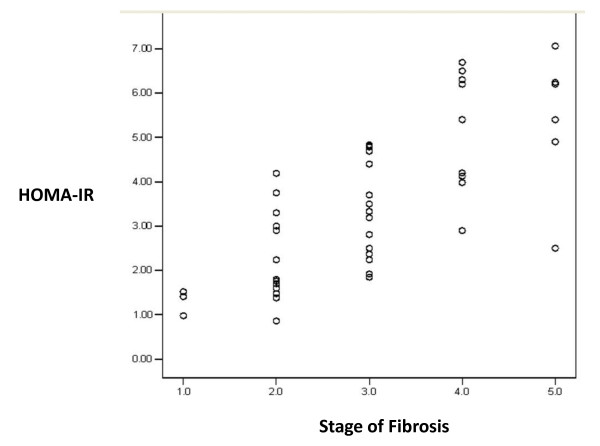
**Correlation between HOMA-IR index and fibrosis among chronic hepatitis C patients**. (r = 0.743, p < 0.001).

**Figure 2:**
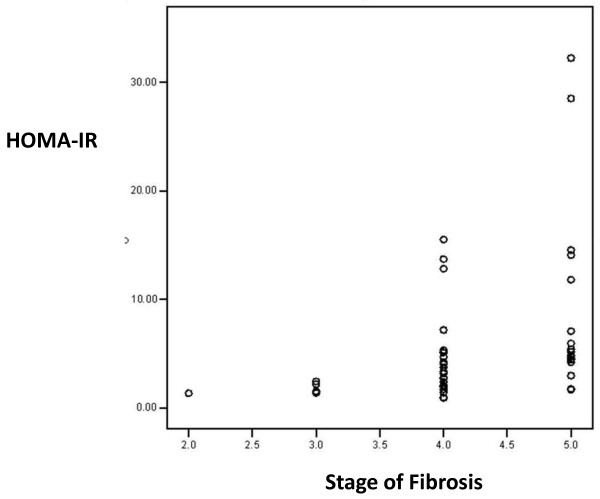
**Correlation between HOMA-IR index and fibrosis among hepatocellular carcinoma patients**. (r = 0.473, p = 0.001).

Multiple linear regression analysis showed that HOMA-IR and INR are the only independent predictors of fibrosis in CHC patients after adjustment (of age, AST, total bilirubin, albumin, glucose, α-fetoprotein, platelets, GGT, fasting insulin, HbA1c, cholesterol and triglycerides). In HCC patients, HbA1c, triglycerides and total bilirubin are independent predictors of fibrosis after adjustment (of age, AST, INR, albumin, glucose, α-fetoprotein, platelets, GGT, fasting insulin, HOMA-IR and cholesterol) Table 3. Correlation between HOMA-IR index and grades of fibrosis in CHC and HCC groups are shown in Figures 1 and 2, respectively.

**Table 3:** Multivariate analysis of factors associated with fibrosis in CHC and HCC patients

Factor	Unstandardized coefficients		t	p-value
	B	Std.error		
**CHC**				
HOMA-IR	0.326	0.087	3.733	**0.001**
INR	1.806	0.812	2.225	**0.031**
**HCC**				
HbA1c	0.122	0.04	3.045	**0.004**
TG	0.006	0.003	2.331	**0.024**
T.Bil	0.182	0.08	2.263	**0.028**

HOMA-IR, Homeostasis Model for assessment of insulin resistance; INR, International normalization ratio (for blood clotting); HbA1c, glycosylated hemoglobin; TG, triglycerides; T.Bil, total bilirubin

### Viral load and insulin resistance

**Figure 3:**
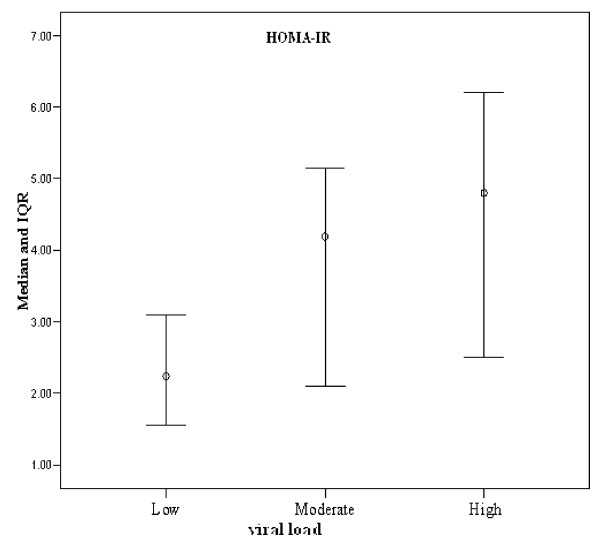
**Plot of insulin resistance (median and IQR of HOMA-IR) associated with different viral loads in chronic hepatitis C group**.

Analysis of HCV viral load with HOMA-IR showed that in the CHC group, both moderate and high viral load were significantly associated with higher values for HOMA-IR compared to those with lower median viral load (4.19, 5.15 vs 1.85, p < 0.001) Figure 3. Similar differences were not observed in HCC patients.

### HCC development

Multivariate logistic regression analysis showed that fibrosis was the only independent predictor factor for risk of HCC development (OR: 21.64, 95%CI: 1.81-259.09, p = 0.02), after adjustment of (age >56y, HBA1c >7%, AST >40, TBil >1, GGT >50, INR>1) (Table 4**)**.

**Table 4:** Multivariate analysis of factors associated with risk of HCC development

Factor	P	Odds ratio	95.0% C.I	
Age>57	0.77	0.83	0.24	2.88

HbA1c>7	0.20	.34	0.07	1.74

AST>40	1.00	7.26	0.00	

TBil>1	1.00	∞	0.00	

GGT>50	1.00	∞	0.00	

INR>1	1.00	∞	0.00	

**Fibrosis (III-IV)**	**0.02**	21.64	1.81	259.09

HbA1c, glycosylated hemoglobin; AST, aspartate aminotransaminase; TBil, Total bilirubin; GGT, gammaglutamyl tranferase; INR, International normalization ratio (for blood clotting).

### Obesity

**Figure 4:**
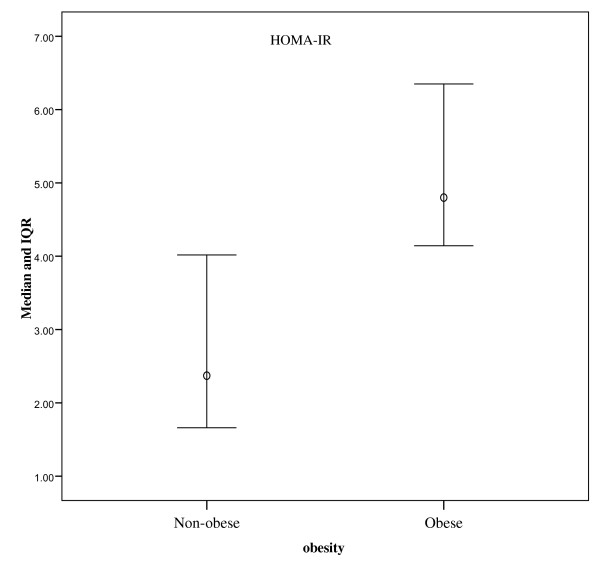
**Plot of median and IQR of HOMA-IR index values among obese (BMI>25) and non-obese subjects**. Difference was significant (p = 0.001).

Obesity was significantly associated with higher HOMA-IR index value in both CHC and HCC patients (p < 0.001). Obese patients (CHC and HCC) showed significantly higher frequency of high viral load when compared to non-obese patients (p = 0.001) Figure 4.

Multiple linear regression analysis showed that fasting insulin and cholesterol are independent predictors for obesity among CHC and HCC patients (OR: 1.663, p < 0.001, OR: 1.052, p < 0.001 respectively) (Table 5).

**Table 5:** Comparison between obese and non-obese patients (CHC and HCC)

Variables	Obese N = 42 N(%)	Non-obese N = 58 N(%)	P-value
Age	60 (36-76)	57(34-73)	0.168
**Biochemical tests *Median(Range)***,			
ALT (IU/L)	60 (36-180)	60(25-103)	0.817
AST (IU/L)	93.5(34-310)	90(25-300)	0.115
T. Bil (mg/dL)	2.1(0.6-4.5)	1.9(0.5-6)	0.092
Albumin (g/dL)	3.1(1.6-4.2)	3.2(1.6-4.2)	0.262
INR	1.2(0.8-1.5)	1.1(0.7-1.5)	**<0.001**
GGT (IU/L)	83.5(25-560)	71(15-500)	**0.043**
Fasting Glu (mg/dL)	190((96-890)	150 (76-870)	**<0.001**
Fasting Ins (IU/mL)	10(6-14)	7.6 (3.5-15)	**<0.001**
HbA1c (**%**)	8.6(5.2-12)	6.6 (3.2-13)	**<0.001**
HOMA-IR	4.8(1.66-28.5)	2.37(0.86-32.22)	**<0.001**
Plt (x10^9^/L)	140(50-250)	140 (60-300)	**0.018**
Cholesterol (mg/dL)	220(160-300)	187 (110-250)	**<0.001**
TG (mg/dL)	212(150-300)	180 (87-220)	**<0.001**
**Tumor marker median(range)**			
Serum AFP (ng/mL)	115(6-1060)	181(5-560)	0.229
**HCV RNA (viral load copies/IU/L) N(%)**			
Low (**<**100 × 10^3^)n = 21	2(4.8)	19 (32.8)	**0.001**
Moderate (100-1000 × 10^3^) n = 56	25(59.5)	31 (53.4)	
High (>1000 × 10^3^) n = 23	15(35.7)	8 (13.8)	**0.001**
**Stage of Fibrosis N(%)**			
I+II (n = 17)	2 (12)	15 (88)	**0.02**
III+IV+ V (n = 83)	40 (48)	43 (52)	

ALT, alanine aminotransferase; AST, aspartate aminotransferase, INR, International normalization ratio (for blood clotting); GGT, gammaglutamyl transferase; Glu, glucose; Ins, insulin; HbA1c, glycosylated hemoglobin; HOMA-IR, Homeostasis Model for Assessment of Insulin Resistance; Plt, platelet count; TG, triglycerides; AFP, alpha fetoprotein;

### CHC and HCC with high HOMA-IR index

Table 6 shows a comparison of CHC and HCC patients demonstrating HOMA-IR > 4. The strong similarities of these variables suggest that HCV-4, like HCV genotypes 1 and 2, leads to fibrosis rather than the steatosis seen with genotype 3.

**Table 6:** Comparison between CHC and HCC regarding HOMA-IR index >4 and high risk variables

Variables	CHC HOMA-IR >4 20(40%)	HCC HOMA-IR >4 26(52%)
**Age**		
*>*57 years	13(65)	17(65)
**Male gender**:	14(70)	17 (65)
**Obese(>25 kg/m^2^)**	16 (80)	17(65)
**LFT ***(Mean ± SD)*		
ALT (>37 IU/L)	18(90)	24(92)
AST (>40 IU/L)	18(90)	26(100)
T. Bil (> 1 mg/dL)	13(65)	26(100)
		
INR (> 1-)	18(90)	26(100)
GGT (>50 IU/L)	17(85)	26(100)
**Biochemical tests**		
Fasting glucose (>110 mg/dL)	20(100)	26(100)
Fasting insulin (>6IU/L)	20(100)	26(100)
HbA1c (>7%)	19(95)	25(96)
		
Cholesterol **(>**200 mg/dL)	14(70)	15(58)
		
**TG**(> 150 mg/dL)	20(100)	25(96)
**Tumor marker**		
Serum AFP (>10 ng/mL)	20(100)	26(100)
**HCV RNA (viral load) N(%)**		
Moderate	12(60)	19(73)
High	7(35)	7(27)
**Stage of Fibrosis**		
III+IV	19(95)	26(100)

## Discussion

This study has provided valuable cues with respect to the potential role of insulin resistance in the pathological consequences of infection with HCV genotype 4 in a population notable for high rates of HCV infection and obesity. Previous studies suggest a strong synergistic effect of metabolic factors and viral hepatitis in HCC development in HCV-infected patients [20,21]. In contrast to a previous report which found association of IR, regardless of diabetes, with development of HCC [11], the present study showed no significant difference in HOMA-IR values, diabetes and insulin levels between CHC and HCC patients. This discrepancy might be due to confounding factors such as:

i) High prevalence of HCV-4 infection; in high prevalence areas the incidence of HCC is heavily influenced by virus-related characteristics that confound other risk factors for HCC, blurring their effect [22]. This may indicate that the effect of IR on the risk of HCC is modified by HCV infection.

ii) High prevalence of obesity among our patients without significant difference between CHC and HCC. It has been documented that obesity is an independent risk factor for IR in HCV and HCC [23].

iii) The number of participants in this study may not have been sufficient to reveal small differences in prevalence in the studied population

In the present study, the frequency of conspicuous insulin resistance (HOMA-IR >4) was 40% among CHC patients. This finding is in agreement with Harrison and co-workers [24], who reported that 30 to 70% of CHC patients display some evidence of IR. These results have suggested the occurrence of IR at early stage(s) of chronic HCV infection irrespective of the severity of liver disease and thus the possible role of IR as a metabolic factor that increases risk of HCC development [25]. It is noteworthy that there are a variety of plausible direct effects of HCV in modulating insulin signaling and molecular pathways involved in IR development in hepatocytes [26,27]. In turn, hyperinsulinemia that results from IR stimulates growth of HCC and inhibits apoptosis in diabetic patients [10,25,28].

It is very likely that a high prevalence of diabetes in our CHC group (96%) may be a risk factor for HCC development [25,29] but this effect may be confounded by the underlying liver disease [22]. Veldt and co-workers [25] have reported that the 5 year risk of developing HCC is 11.4% for patients with both diabetes mellitus and CHC with advanced fibrosis while patients without diabetes have lower risk of HCC (occurrence of HCC in 5% after 5 years).

Multivariate analysis showed that HOMA-IR values and INR were independently associated with fibrosis in CHC patients, but not in the HCC patient group. This is in accord with our hypothesis that early occurrence of IR may hasten progression of fibrosis to cirrhosis which may culminate in HCC development. This is consistent with recent reports, including a Japanese cohort study [30,31].

It has been reported that the mean IR index increases with stage of fibrosis and may help in differentiating fibrosis stages [32]. This was observed in our results (Figure 1) where HOMA-IR was significantly associated with high grades of fibrosis in CHC patients (p < 0.001). IR promotes fibrosis progression in the liver of patients with HCV through development of hepatic steatosis, hyperleptinemia, increased TNF production and reduced expression of peroxisome proliferator activated receptors (PPAR ץ receptors) [27,33]. High levels of insulin and glucose could promote fibrogenesis by stimulating release of connective tissue growth factor from hepatic stellate cells [34]. The present study did not investigate steatosis as a possible link between IR and fibrosis.

In our HCC patient group, HbA1c, triglycerides and total bilirubin levels were found to be independently associated with fibrosis. This finding was not observed in previous studies. Furthermore, multivariate analysis showed that fibrosis was the only independent predictor for risk of HCC development after adjustment of age and other markers of advanced liver disease (Plt, Bil, albumin, INR AST, GGT). These results confirm a distinct role of diabetes in HCC development [25]; in tumorigenesis, transformed cells require more glucose [35].

It is well established that IR is associated with abdominal obesity, elevated blood cholesterol and hypertension [36]. In the present study, obesity was found significantly associated with IR (higher HOMA-IR index value) in both CHC and HCC patient groups (p < 0.001 compared to non-obese patients). Obesity may directly lead to a state of chronic inflammation with consequent increases in the expression of signaling molecules, some of which (for example, NFKB, and fibroblast growth factor) are thought to be involved in carcinogenesis [37,38]. Chen and co-workers [21] have reported that although overweight itself did not appreciably increase risk of HCC, a combination of high BMI and diabetes showed a synergistic effect. Our results confirmed a synergistic effect of obesity when combined with high level of insulin resistance (HOMA-IR index) in risk of HCC development associated with HCV-4 infection.

Multivariate analysis in the present study showed that fasting insulin and cholesterol were independent predictors for obesity. This readily explains the prevalence of obesity among our patients. Type 2 diabetes is a major health problem in the Eastern Mediterranean Regional Office (EMRO) region and many studies have established a panel of risk factors associated with IR and type 2 diabetes; family history, body fat distribution, age, gender, smoking and physical activity. Obesity and IR are strong cofactors of HCV-related liver disease and predict lack of response to treatment [39].

Division of patient groups using age of >57 revealed that most of our HCC patients were over 57 years in age and the number in this age group showed significant difference when compared to CHC patients (p = 0.04). In univariate analysis, IR was correlated with age in CHC patients. It has been suggested that age is associated with a decline in mitochondrial function which could contribute to IR [40]. However, this relationship disappeared in multivariate analysis; this indicates that age is one of the confounding factors for development of IR [31].

Our HCC patients showed higher frequency of moderate viral load than patients with CHC infection; a result in accord with that reported by Hsu [33]. Our patients (CHC or HCC) showed that moderate or high viral load was significantly associated with high levels of insulin resistance (high HOMA-IR index) compared to those with low viral load (median 4.19, 4.80 vs 2.24, p < 0.001). Interestingly, on analysis of correlation between viral load and HOMA-IR in our CHC and HCC groups separately, it was apparent that in the CHC patient group, cases with moderate and high viral load were significantly associated with higher HOMA-IR index compared to those with lower viral load (4.19, 5.15 vs 1.85, p < 0.001) while in HCC patients, moderate and high viral loads were not significantly associated with higher HOMA-IR values (median 4.19, 4.67 vs 3.06 p = 0.70 compared to cases with lower viral load). This observation is readily rationalized by a role of HCV infection in production of IR before cirrhosis and HCC development. This supports the hypothesis that a modulating effect of HCV on insulin signaling pathways plays a role in a separate pathway involved in the process of hepatocarcinogenesis [41].

The association of HOMA-IR with viral load is an important finding of the study. The patient with HCV infection can present with IR due to metabolic and to viral factors; the molecular bases of these effects have been subject to intense research scrutiny though the relative importance of some of these interrelated effects to the clinical picture remains to be fully determined. Chronic inflammation as a non-specific consequence of hepatic inflammation due to HCV infection leads to IR associated with metabolic liver disease through increased levels of IL-1, TNF-α, IL-6 and leptin, and reduced levels of adiponectin. This will complement obesity effects which involve simulation of inflammatory mediator IkB kinase β and phosphorylation of IRS-1 [26].

Multiple pathways for direct effects of the virus on IR have been identified. These include impairment of insulin signaling through stimulation of proteosomal inactivation of IRS-1 and IRS-2, viral induction of excess TNF giving enhanced production of suppressor cytokine signal proteins (SOCS) in a genotype specific manner [42] which act on the Akt pathway (also down-regulated by HCV-stimulated PP2A activity) giving impairment of GLUT-4 glucose transporter activation [43]; and action of HCV core protein as an inhibitor of PPAR α (peroxisome proliferator-activated receptors) [44]. In addition, HCV NS5a protein induces mitochondrial ROS (reactive oxygen species) production of which activate release of a cascade of inflammatory mediators through activation of nuclear factor-κB (NF-κB) and HCV NS3, and induce endoplasmic reticulum stress signals. Progressive fibrosis induces IR due to impairment in insulin clearance with resulting insulin levels [17,31].

## Conclusions

Insulin resistance may be induced by HCV infection irrespective of the severity of liver disease; insulin resistance starts at an initial phase of HCV infection and facilitates the progression of fibrosis and development of HCC.

In chronic hepatitis C infection, HCV viral replication *per se *may be dominant in the overall process of hepatocarcinogenesis associated with HCV infection. This study complements studies of HCV infection in other populations and indicates that in the Egyptian population suffering from a high burden of hepatitis C genotype-4 virus the strikingly high rates of hepatocarcinogenisis result from a combination of this direct viral effect and the influence of an array of metabolic factors resulting from virus-induced insulin resistance.

Our results lay a foundation for a follow up cohort study with larger sample size and including diabetic and non-diabetic CHC patients with and without fibrosis that would have value to illustrate the role of IR as a separate metabolic factor for HCC development and enable investigation of the role of other individual metabolic signals such as adiponectin, and leptin which may have particular involvement in hepatocarcinogenesis in patients infected with HCV-4. A better understanding of IR involvement in HCV-4 mediated HCC may point to potential for benefit of therapeutic interventions aimed at control of IR, and IR-associated signals, in these patients.

## Competing interests

The authors declare that they have no competing interests.

## Authors' contributions

AAM performed the research; SAL developed the plan of the study and wrote the paper; JC analyzed the molecular data and evaluated and edited the draft manuscript; AGMH contributed analytical tools and reagents and IS collected samples, gathered clinical data and contributed to the drafting data tables.

All authors have read and approved the final manuscript
